# Leaflet Lengths and Commissural Dimensions as the Primary Determinants of Orifice Area in Mitral Regurgitation: A Sobol Sensitivity Analysis

**DOI:** 10.3390/bioengineering13010097

**Published:** 2026-01-14

**Authors:** Ashkan Bagherzadeh, Vahid Keshavarzzadeh, Patrick Hoang, Steve Kreuzer, Jiang Yao, Lik Chuan Lee, Ghassan S. Kassab, Julius Guccione

**Affiliations:** 1 Department of Mechanical Engineering, Michigan State University, East Lansing, MI 48823, USA; bagherza@msu.edu (A.B.); lclee@msu.edu (L.C.L.); 2 3DT Holdings LLC, San Diego, CA 92121, USA; vkeshavarzzadeh@3dtholdings.com (V.K.); gkassab@calmi2.org (G.S.K.); 3 School of Medicine, University of California San Francisco, San Francisco, CA 94117, USA; patrick.hoang@ucsf.edu; 4 Exponent, Inc., Menlo Park, CA 94025, USA; skreuzer@exponent.com; 5 Dassault Systèmes, Johnston, RI 02911, USA; jiang.yao@3ds.com; 6 California Medical Innovations Institute, San Diego, CA 92121, USA

**Keywords:** mitral valve, geometric parameters, gaussian process, global sensitivity analysis

## Abstract

Mitral valve orifice area is a key functional metric that depends on complex geometric features, motivating a systematic assessment of the relative influence of these parameters. In this study, the mitral valve geometry is parameterized using twelve geometric variables, and a global sensitivity analysis based on Sobol indices is performed to quantify their relative importance. Because global sensitivity analysis requires many simulations, a Gaussian Process regressor is developed to efficiently predict the orifice area from the geometric inputs. Structural simulations of the mitral valve are carried out in Abaqus, focusing exclusively on the valve mechanics. The predicted distribution of orifice areas obtained from the Gaussian Process shows strong agreement with the ground-truth simulation results, and similar agreement is observed when only the most influential geometric parameters are varied. The analysis identifies a subset of geometric parameters that dominantly govern the mitral valve orifice area and can be reliably extracted from medical imaging modalities such as echocardiography. These findings establish a direct link between echocardiographic measurements and physics-based simulations and provide a framework for patient-specific assessment of mitral valve mechanics, with potential applications in guiding interventional strategies such as MitraClip placement.

## 1. Introduction

Severe mitral regurgitation (MR) initially increases left atrial volume and pressure, and over time imposes a chronic volume load on the left ventricle due to regurgitant flow returning during diastole, leading to progressive ventricular dilation and eventual heart failure (HF). Patients with severe MR are not always suitable candidates for surgical repair or replacement. Advanced age, frailty, reduced left ventricular function, significant comorbidities such as chronic kidney disease or pulmonary hypertension, and prior cardiac surgery may substantially increase operative risk. In addition, patients with functional MR secondary to left ventricular remodeling often derive limited benefit from surgical intervention alone. These factors have motivated the development and adoption of transcatheter edge-to-edge repair techniques as less invasive alternatives for selected high-risk patient populations.

According to current clinical guidelines, severe mitral regurgitation is defined using an integrated echocardiographic assessment, with a key quantitative criterion being an increased effective regurgitant orifice area, typically ≥ 0.4 cm^2^ for primary MR, along with supporting measures such as regurgitant volume, vena contracta width, and associated chamber remodeling [1]. For patients with severe MR who are poor candidates for surgical repair, the MitraClip (MC; Abbott Vascular, Santa Clara, CA, USA) offers a minimally invasive alternative that restores leaflet coaptation and reduces regurgitant flow. Clinical trials such as EVEREST and COAPT have demonstrated that MC therapy can improve symptoms, enhance functional capacity, and reduce HF hospitalizations in appropriately selected patients [2,3,4].

Despite these successes, outcomes after MC implantation remain variable, with some patients exhibiting incomplete MR reduction or recurrent regurgitation. This variability reflects the multifactorial nature of procedural success and underscores the need for improved patient selection and procedural planning strategies [5].

A major contributor to this variability is the heterogeneity in mitral valve (MV) anatomy across patients [6]. Variations in annular dimensions [7], leaflet morphology [8], and overall valve geometry [9] can significantly influence clip placement, leaflet grasping, and the resulting post-clip orifice area [10,11]. In patients with advanced MR, these geometric features are often more distorted, further complicating procedural decisions. Additionally, identifying the dominant MR jet (crucial for effective MC placement) can be challenging in certain cases, further increasing the difficulty of achieving optimal outcomes [12,13].

Computational modeling has emerged as a powerful approach for investigating mitral valve biomechanics and guiding transcatheter interventions. Finite element (FE) analysis enables simulation of leaflet mechanics, annular motion, and device–tissue interaction under physiological conditions, providing insights that are difficult to obtain experimentally or clinically. Earlier studies demonstrated how modeling the nonlinear tissue behavior and annular dynamics can reproduce realistic valve function and identify key mechanical contributors to repair performance [14,15]. These advances established the foundation for translating FE analysis into a clinically relevant tool for evaluating procedural outcomes.

Building on this progress, computational modeling now provides an opportunity to systematically examine how individual geometric factors influence MC performance under controlled, patient-specific conditions. Recent studies have expanded FE modeling toward comprehensive, patient-derived frameworks that assess post-intervention hemodynamics and valve function [16,17,18].

While these advances in modeling have enabled detailed investigation of how mitral valve anatomy influences output metrics, the high-fidelity physics-based simulations remain computationally expensive, limiting their utility for large-scale exploration of geometric variability. In this work, we focus on quantifying how key anatomical parameters impact the orifice area (OA), an important determinant of mitral valve function. Because evaluating thousands of physics-based simulations is impractical, we construct a surrogate model that efficiently maps a 12-dimensional geometric parameterization of the leaflets and annulus to the resulting OA. Gaussian Process regression is particularly suitable for this task because unlike many machine learning approaches, its performance is less sensitive to the number of dimensions, making it well suited to our parameter space. Once trained, the GP surrogate enables rapid generation of a large synthetic dataset spanning the full physiologic range of geometries. This dataset forms the basis for a global sensitivity analysis using Sobol indices, allowing us to numerically estimate the relative influence of each geometric feature and identify the parameters that most strongly govern OA.

Prior studies have applied similar simulation, surrogate, and sensitivity pipelines. For example, work on left ventricular passive mechanics using GP models and Sobol analysis [19] or recent studies that relate mitral valve anatomy to clinical markers via surrogate modeling [20], but these efforts typically rely on fixed or simplified valve geometries and do not include an in-depth representation of leaflet and annular shape. In contrast, our framework integrates a detailed geometric parameterization with a data-efficient surrogate model and a rigorous global sensitivity analysis, providing a comprehensive characterization of how anatomical variability drives changes in OA. Importantly, the parameters identified as most influential in our study correspond directly to quantities measurable on standard echocardiography. Prior work has highlighted the importance of patient-specific modeling for improving device guidance and characterizing valve biomechanics [21,22]. Building on this foundation, our framework provides a clear path toward clinical translation and is well suited for patient-specific assessment and procedural planning.

This paper is organized as follows. Section 2 describes the simulation setup, the geometric parameters, and the methodology for GP regression and Sobol sensitivity analysis. Section 3 presents the results, including the performance of the GP regression model, its comparison with linear regression, and the Sobol sensitivity analysis that identifies four influential parameters. Section 4 provides concluding remarks, and Section 5 discusses the limitations of this work and outlines potential future directions.

## 2. Materials and Methods

In this section, we describe the simulation setup for finding the orifice area associated with different geometric parameters as well as our description on Gaussian Process regression and Sobol sensitivity analysis as utilized in this work.

### 2.1. Simulation Setup

To generate anatomically realistic MV models, we use the 3DEXPERIENCE Platform (Dassault Systèmes) to construct the leaflet morphology and annular geometry, followed by mesh generation. We then import the resulting 3D MV geometries into Abaqus for FE simulation of MV mechanics. These simulations were based on key geometrical measurements of the MV and were used to compute the OA.

Our FE modeling workflow has been described in previous studies [23,24], and is briefly summarized here. The model parameters used in the simulations are summarized in Table 1 and Table 2. Table 1 lists the geometric and boundary-condition parameters used to construct the mitral valve models, while Table 2 summarizes the leaflet material parameters [25]. Although all parameters were required to construct a physiologically realistic MV model, only 12 geometrical parameters were varied within the specified ranges shown in Table 1, while the remaining parameters were kept constant across all simulations. The twelve geometric parameters included in the model were defined to capture key aspects of mitral valve size, leaflet proportion, and annular geometry.

The anteroposterior diameter (AP_D) represented the annular diameter measured between the A2 and P2 scallops. In the standard anatomical classification of the mitral valve, the anterior leaflet is divided into three scallops. A1 (lateral), A2 (central), and A3 (medial) and the posterior leaflet is divided into P1 (lateral), P2 (central), and P3 (medial). The A2–P2 region corresponds to the central functional zone of leaflet coaptation; therefore, the AP_D measurement reflects the most biomechanically relevant anteroposterior dimension of the annulus.

The intercommissural diameter ratio (IC_D_R) quantified the transverse width of the annulus by normalizing the intercommissural diameter to the AP_D. Leaflet proportions were described by the anterior leaflet length ratio (AL_R), defined as anterior leaflet length divided by AP_D, and the posterior leaflet length ratio (PL_R), defined as posterior leaflet length divided by the remaining AP dimension after subtracting the anterior leaflet length (PL_R = PL/[AP_D − AL]). The commissure length ratio (CL_R) captured commissural geometry as the ratio of commissure length to the posterior leaflet length minus the posterior annular height (CL_R = CL/[PL − PH]). The anterior leaflet bluntness (Ant Blunt) quantified how rounded or flat the anterior leaflet free edge was.

**Figure 1:**
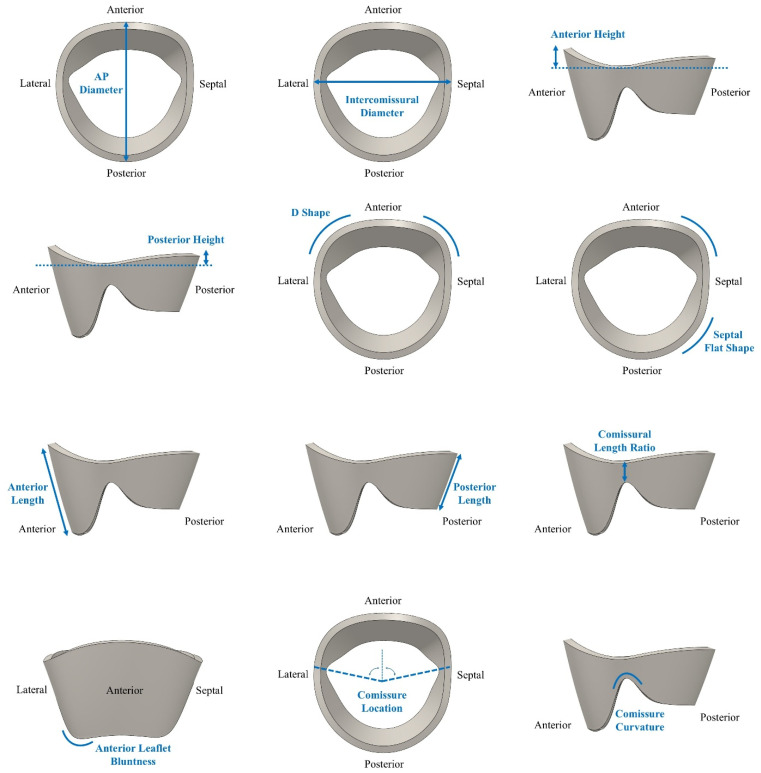
MV leaflet geometry and the 12 key characteristic parameters.

Anterior height (AH) and posterior height (PH) measured the vertical height of the anterior and posterior annulus relative to the annular plane. The D shape parameter (D Shape) described the curvature of the anterior annulus, and the septal flatness ratio (SF Shape Ratio) quantified the flatness of the septal annulus normalized to the intercommissural diameter. Finally, commissure location (Com_Loc) captured the top-down position of the commissures, and commissure curvature (Com_Cur) described the curvature of the commissural free edges. A visual representation of the geometrical definition of each leaflet parameter is provided in Figure 1.

**Figure 2:**
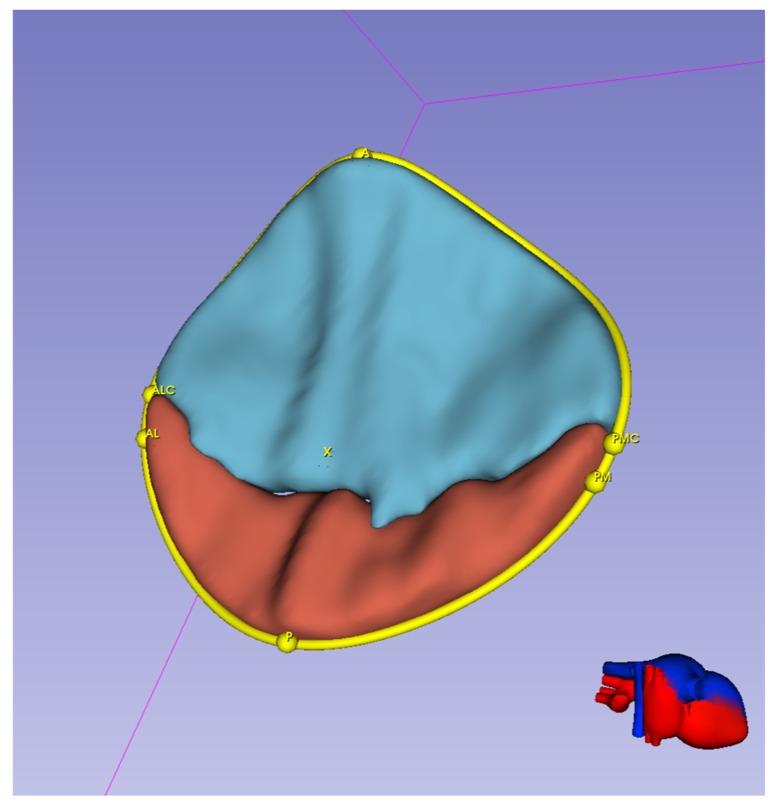
Manual segmentation of mitral valve and identifying landmark points which quantify influential geometric parameters. ALC-PMC quantifies intercommissural diameter and distances AX and PX approximately quantify anterior leaflet length and posterior leaflet length.

To demonstrate the correspondence between influential geometric parameters with dimensions that could be extracted from echo images, we show a segmented mitral valve at the mid-systole where a number of landmark points are specified cf. Figure 2. In this paper, we show that intercommissural diameter, posterior and anterior lengths as well as commissural length are the most influential parameters that represent variability in orifice area. In Figure 2, ALC-PMC quantifies intercommissural diameter and distances AX and PX approximately quantify anterior leaflet length and posterior leaflet length. The commissure length is not visible in this figure; however, we could extract that quantity from segmentation at end-diastole directly. We do not show that quantity in this paper as segmentation at end-diastole requires more careful attempt (as the visibility on the echo is generally lower) and we establish the connection between CL in our computational mesh and CL in echo image in our future work.

Boundary conditions simulated the dynamic motion of the MV annulus during interaction with the left ventricle. Annular motion is prescribed using two kinematic parameters, Annulus_dh and Annulus_dr, which represent the time-varying apico-basal displacement and radial expansion or contraction of the annulus, respectively. In the finite element model, annular nodes are assigned time-dependent displacement boundary conditions synchronized with the cardiac cycle. Specifically, Annulus_dh prescribes displacement of the annulus along the left ventricular long axis to capture systolic annular descent toward the ventricle, while Annulus_dr prescribes in-plane radial deformation to represent annular contraction during systole and expansion during diastole. These boundary conditions are applied uniformly to the annular node set and are independent of leaflet geometry variations.

**Figure 3:**
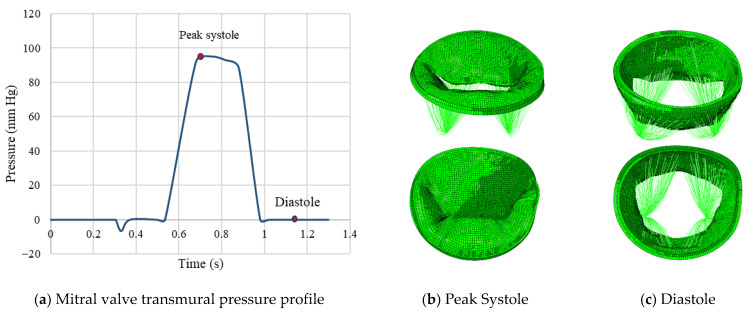
Applied pressure profile throughout the time and corresponding leaflet geometry for systole and diastole phase.

Leaflet tissue material behavior was modeled using the Holzapfel–Ogden hyperelastic constitutive model [26,27], with material parameters summarized in Table 2. Chordae were modeled using truss elements and Ogden constitutive model. The physiological transmural pressure profile applied to the MV leaflets throughout one cardiac cycle is illustrated in Figure 3, along with the corresponding valve configurations at two time points of peak systole and diastole. As shown, a small residual orifice gap remains open during closure, consistent with clinically observed physiological regurgitation.

**Figure 4:**
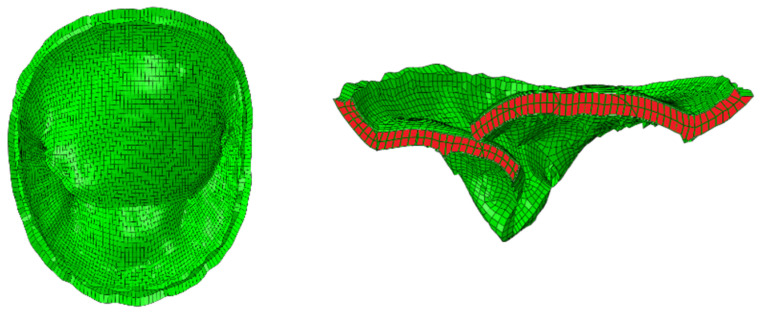
MV at mid-systole from top-view (**left**) and mid-cross section of the anterior and posterior leaflets (**right**).

For the sake of studying the effect of geometric parameters on orifice area, simulations were performed under pre-clip conditions (i.e., we do not perform simulations where effect of clip is considered). We mainly focus on the pre-clip condition in this paper as we believe the effect of geometric parameters on orifice area remain consistent across different conditions, i.e., pre-clip and post-clip analysis. Figure 4 shows the computational mesh of MV associated with the pre-clip condition in different views. It should be noted that we consider orifice area during valve closure in middle of systole.

OA was quantified using the gap-length method, in which the valve was segmented into 40 equally spaced cross-sectional planes spanning the commissure-to-commissure direction. Each plane was oriented perpendicular to the local coaptation line and intersected with the anterior and posterior leaflet surfaces to extract the corresponding leaflet free-edge geometry at that location. For each cross-section, the intersection points were separated into two boundary curves representing the opposing anterior and posterior leaflet edges. The local gap length was defined as the minimum distance between these two curves within the plane, corresponding to the local leaflet separation. If the two curves overlapped or came into contact, the gap length was set to zero to avoid non-physical negative values. The total orifice area was then computed by discrete integration across the valve span, calculated as the sum of the local gap lengths over all cross-sections. This procedure yields a spatially resolved estimate of the orifice area that accounts for non-uniform leaflet separation along the coaptation line.

### 2.2. Gaussian Process Regression

A Gaussian Process is a random process in which the function values at any finite set of inputs are jointly Gaussian. A main component of Gaussian Processes is the kernel matrix, which encodes the relationships between samples [27]. The optimizable kernel parameter, typically a kernel length scale, is chosen to maximize the log marginal likelihood. For GP regression, we consider a vector of length scales within an Automatic Relevance Determination (ARD) kernel of the following form:

$$k\left(x_{i},x_{j}\right)=exp\left(-\frac{1}{2}\sum_{d=1}^{D}\frac{{{(x}_{\left\{i,d\right\}}-x_{\left\{j,d\right\}})}^{2}}{{l_{d}}^{2}}\right)$$

where *D* is the number of variables and $l_{d}$ is the length scale associated with dimension d. It is noted that the number of optimizable parameters is the same as the number of dimensions; therefore, in terms of optimization scale and training simplicity, this approach is comparable to linear regression, which also has a number of unknowns equal to the number of variables. However, GP can effectively describe nonlinear relationships between inputs and outputs, whereas linear regression ceases to be adequate. We provide a comparison in our numerical results to demonstrate the performance of the two approaches.

Once the optimal hyperparameters are obtained, the GP mean prediction is computed as follows:

$$y_{*}=k\left(x_{*},X\right){\left(K+\sigma^{2}I\right)}^{-1}y$$

where y is the vector of ground-truth data, $k\left(x_{*},X\right)$ is the kernel vector evaluated between the unseen input sample and the training samples, and K is the kernel matrix evaluated using the training samples. A small, fixed $\sigma^{2}$ is added to the kernel matrix for numerical stability, which also helps prevent overfitting during GP training.

To build a GP regressor on geometric parameters, we specifically work with unnormalized geometric parameters where we consider AP = AP_D, IC = IC_D_R × AP, AL = AL_R × AP, SF_shape = SF_shape_R × IC, PL = PL_R × (AP − AL), CL = CL_R × (PL − PH) and the rest of parameters {AH, PH, D_shape, Ant_blunt, Com_loc, Com_Cur} are already in their plain form. To study the prediction performance, we mainly consider two numerical measures, coefficient of determination which reads

$$R^{2}=1-\frac{\sum {\left(y-\hat{y}\right)}^{2}}{\sum {\left(y-\bar{y}\right)}^{2}}$$

and mean absolute percentage error (MAPE) expressed as

$$MAPE=\frac{1}{N}\sum \left|\left(\frac{y-\hat{y}}{y}\right)\right|$$

### 2.3. Sobol Sensitivity Index

To evaluate the importance of each geometric parameter, we use the Sobol sensitivity index [28]. The Sobol index quantifies how much variability in each input variable contributes to the variance of the output, in this case, the orifice area. It measures the proportion of output variance explained by each input variable and provides a global assessment of variable importance. By identifying the most influential parameters, one can achieve accurate predictions using only those key inputs.

The first order Sobol index for input variable X_i_ is defined as S_i_ = V_i_/Var(y) where V_i_ is given by

$$V_{i}={Var}_{X_{i}}\;\left(E_{X_{\sim i}}\left[Y|X_{i}\right]\right)$$

To compute *V_i_* in 12 dimensions for each variable we need to consider number of points where at that particular dimension, that number is fixed (assume, for example, X_1_ = 1.85) and the rest of dimensions are varying. We obtain the output at those samples, compute the mean of outputs and then repeat this process for different fixed numbers at the particular dimension (for example, X_1_ = 2.21, X_1_ = 4.25, …). We finally compute the variance of resulting outputs and that gives V_i_ which then is used to compute Sobol index.

## 3. Results

We first present results demonstrating the performance of our GP model and compare it with linear regression. Once the model is established, we use all samples to build the surrogate and perform the sensitivity analysis.

### 3.1. Demonstration of GP Predictive Performance—Comparison with Linear Regression

In this section, we analyze 440 samples obtained from simulations performed in Abaqus. Geometry creation for each sample takes approximately 30 min, and running the simulation requires another 30 min. The samples are initially generated as uniformly distributed within the ranges specified in Table 1, after which we unnormalize the geometric parameters as described in Section 2.2. We use 350 samples for training and 90 for testing. As explained in Section 2.2, the GP is optimized with respect to a length-scale parameter. The initial guess for this parameter is treated as a hyperparameter, and its optimal value is obtained through 5-fold cross-validation. To reduce clutter, we do not show the cross-validation results. Instead, we report the R^2^ and MAPE values on the test set. To obtain a more stable estimate of test error, we repeat the training–testing process over five random 350-90 splits of the 440 data points.

We treat the linear regression model as similarly as possible to the GP to ensure a fair comparison. That is, we also consider five random 350-90 splits for training and testing. It should be noted that linear regression has no hyperparameters; therefore, no cross-validation is required for that case.

**Figure 5:**
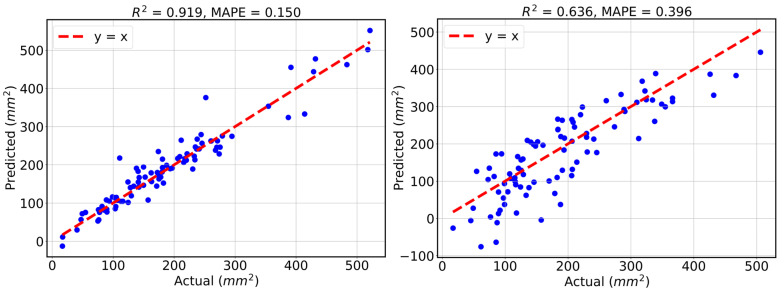
R^2^ and MAPE results on GP regression (**left**) and linear regression (**right**) associated with one experiment of 5 random splits of train and test samples.

Figure 5 shows the results from one of the five random splits. The mean R2 and MAPE values for the GP and linear regression models are reported as (0.920, 0.156), (0.636, 0.377). From these results, as well as the visual comparison from one representative experiment, it is evident that the GP provides more accurate predictions in this 12-dimensional space. This is expected, as the relationships between geometric parameters and orifice area are inherently nonlinear.

### 3.2. Sobol Index Results

Once we confirm the suitability of the surrogate model using the optimal hyperparameter identified in the previous section, we rebuild the surrogate using all 440 samples with the same optimal hyperparameter. This full surrogate is then used to generate Monte Carlo samples for computing the Sobol indices. To this end, we generate 1000 uniform samples of the geometric parameters (first in normalized form, then unnormalized). For each dimension, we repeat the value of that dimension 1000 times while allowing the remaining 11 parameters to vary. This procedure results in a total of 1 million samples, which are used to compute the Sobol sensitivity indices.

**Figure 6:**
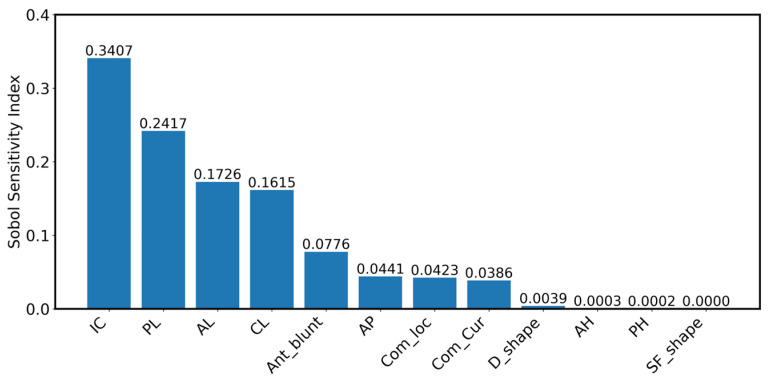
Result of Sobol sensitivity analysis on effect of geometric parameters on orifice area. InterCommissural diameter, Posterior length, Anterior length, and Commissure length are identified as the most important descriptors of mitral valve geometry.

Figure 6 shows the Sobol indices for the one-dimensional variables. As seen, InterCommissural diameter (IC), Posterior length (PL), Anterior length (AL), and Commissure length (CL) have the largest contributions to the variation in orifice area. The first parameter, IC, forms the base for the orifice area, so it is expected that it has the highest influence.

Once we identify the most influential parameters, which in our analysis are IC, AL, PL, and CL, we construct the probability density function (PDF) of the orifice area in three cases: (1) Simulation results, (2) GP with all parameters varying, and (3) GP with only the four influential parameters varying while the remaining eight parameters are fixed at their mean values. We compute the total variation to estimate the differences between Sim-GP12, Sim-GP4, and GP4-GP12. The total variation measure defined as

$$TV\left(p,q\right)=\frac{1}{2}\int \left|p\left(x\right)-q(x)\right|dx$$

between two pdfs (p,q) provides a normalized estimate that can be more easily interpreted as a measure of error or distance.

**Figure 7:**
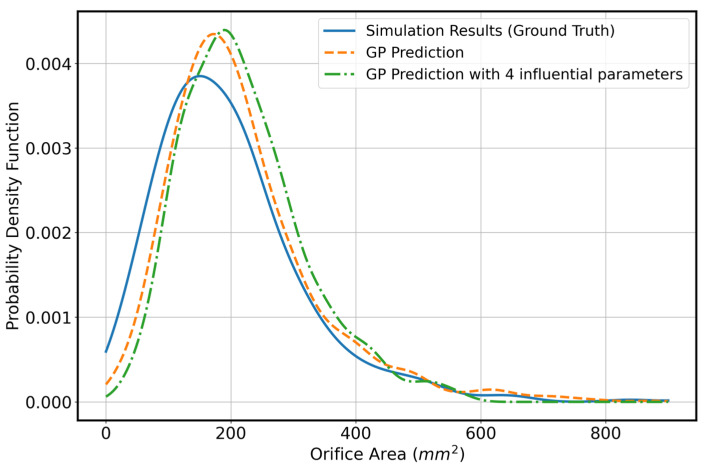
Probability density function for (1) ground truth orifice are (2) orifice area from GP regressor with all 12 parameters varying and (3) orifice area from GP regressor with only 4 influential parameters varying. The total variation distance between these distributions is deemed to be small which indicates that using only 4 influential parameters would be sufficient to describe geometry of mitral valve for predicting the orifice area.

Figure 7 shows the three distributions, and the numerical values of total variation are 0.083, 0.137, 0.073 for Sim-GP12, Sim-GP4, and GP4-GP12. Based on these results, the differences between the predictions from the GP using only the four influential parameters and both the ground truth and the GP with all parameters varying are relatively small. Therefore, describing the mitral valve geometry using these four influential parameters is sufficiently accurate.

## 4. Discussion

In this study, we identified four influential geometric descriptors of the mitral valve, intercommissural diameter (IC), posterior leaflet length (PL), anterior leaflet length (AL), and commissural length (CL) that have the greatest impact on the variability in orifice area. For this study, we developed a GP surrogate model to characterize the mitral valve orifice area based on anatomical parameters. The GP model showed strong agreement with physics-based simulation results and outperformed a linear regression baseline. We performed Sobol sensitivity analysis to identify influential parameters that dominate the variability in orifice area. Notably, all four influential parameters can be extracted from echocardiographic imaging.

Because performing a robust sensitivity analysis requires a large number of samples, we leveraged the GP surrogate to efficiently generate Monte Carlo samples, enabling accurate computation of Sobol indices. Using these influential parameters, we constructed probability distributions of orifice area under three conditions: Simulation results, GP with all 12 parameters varying, and GP with only the four influential parameters varying. The total variation distances among these distributions were small, indicating that the four-parameter GP surrogate captures the essential geometric variability with sufficient accuracy.

We note that the Sobol sensitivity analysis in this study focuses on first-order indices, which quantify the contribution of each individual geometric parameter to the variance of the mitral valve orifice area. While established sampling approaches exist to ensure numerical stability and computational efficiency, and interaction effects can be estimated directly or via total-order indices, our approach follows a standard procedure for generating a large number of Monte Carlo samples, focusing solely on first-order indices for univariate parameters. By leveraging a Gaussian Process surrogate, we can efficiently generate these samples, mitigating the need for more complex sampling schemes. We also note that the joint effects are generally small in global sensitivity analysis. Our primary goal is to identify the most influential univariate parameters, as these directly guide which geometric features should be prioritized for extraction from echocardiographic images. We emphasize that first-order indices provide clear guidance on parameter importance and are sufficient for the practical, image-based focus of this work.

Overall, these results suggest that mitral valve geometry can be represented efficiently using a reduced set of parameters without significant loss of accuracy, supporting the use of compact geometric descriptors in downstream biomechanical modeling.

### Limitations of Study and Future Directions

While this study provides a structured framework for characterizing mitral valve orifice area using a reduced set of geometric parameters, some limitations remain. First, it should be noted that this study is limited to the pre-clip condition, and the sensitivity rankings presented pertain to this baseline state. While post-clip mechanics may change due to device–tissue interaction, the orifice area as a statistical measure is expected to maintain a similar dependence on the twelve geometric parameters. Given the orifice area as a response surface function of the twelve geometric parameters, the overall form of its statistical dependence on the input parameters is expected to remain unchanged between pre-clip and post-clip conditions, since in both cases we analyze the influence of the same twelve parameters on orifice area. If we considered different output measures, such as mean von Mises stress, we would expect the ranking of influential parameters to differ from those observed for orifice area. Even in that case, however, we do not believe this would change the major conclusions of the study. Second, we focus on twelve key geometric parameters of the mitral valve, while other simulation parameters, including material properties and papillary muscle positions, were held constant. We note that variations in chordae length, as well as papillary muscle displacement, can interact with these anatomical parameters and potentially influence the orifice area and the ranking of influential variables. A systematic exploration of all such interactions would require an expanded simulation framework beyond the scope of the present work. We have therefore limited our analysis to the twelve parameters that can be most reliably and practically obtained from echocardiographic images. Third, the computation of orifice area remains an evolving topic. In this work, we relied on an in-house algorithm that was previously available to us. Future efforts will revisit the orifice area computation and explore new methods for estimating orifice area directly from medical images. Fourth, several biomechanical extensions to the finite element formulation remain promising. A coupled left ventricle–mitral valve (LV–MV) model would enable simultaneous simulation of ventricular contraction, annular deformation, and leaflet coaptation, providing a more complete picture of valve mechanics. Incorporating fluid–structure interaction would further allow joint modeling of leaflet motion and intracardiac flow, improving assessment of post-clip hemodynamics. Extending the framework to the analysis of the tricuspid valve is also a potential future direction [29,30].

Additional directions include the systematic treatment of uncertainty. Quantifying how uncertainties in material properties, geometric measurements, and boundary conditions propagate through the model remains an important scientific challenge and an area in which relatively little work has been performed in mitral valve simulation. Our future work will include a systematic investigation of uncertainty, with a focus on post-clip cases, to assess how uncertainties impact the optimal placement of the MitraClip. Finally, further integration of image-derived anatomy (particularly parameters that can be measured noninvasively) may improve realism and expand opportunities for model personalization without requiring assumptions beyond what can be inferred from standard clinical imaging.

Together, these directions offer a pathway toward increasingly comprehensive, physiologically grounded simulations that can deepen mechanistic understanding of mitral valve behavior and device–tissue interactions across a broad anatomic spectrum.

## 5. Conclusions

Beyond the methodological advances, the findings of this study have direct implications for clinical assessment and transcatheter intervention planning in patients with severe mitral regurgitation. Current clinical guidelines from the European Society of Cardiology (ESC) and the American Heart Association (AHA) emphasize comprehensive echocardiographic evaluation for grading mitral regurgitation severity and for determining anatomical suitability for intervention, including transcatheter edge-to-edge repair [31,32]. Within this framework, the identification of the most influential geometric parameters as determinants of orifice area provides a mechanistic basis for prioritizing specific anatomical measurements during pre-procedural imaging assessment.

In current clinical practice, anatomical suitability for transcatheter edge-to-edge repair relies on a combination of qualitative assessment and threshold-based criteria, which can be challenging in patients with complex or borderline valve anatomy. The reduced set of influential geometric parameters identified here offers a strategy toward more quantitative, anatomy-driven stratification of patients, potentially improving consistency in patient selection and procedural planning. Moreover, by linking routinely measurable imaging features to a functional outcome metric through a surrogate modeling framework, this approach supports future integration into clinical decision-support tools consistent with ESC and AHA guidelines, with the potential to improve procedural planning, and transcatheter intervention strategies.

## Figures and Tables

**Table 1:** Model parameters and their values with description.

Category	Parameter	Value	Description
Leaflet	AP_D	[24.1, 45.2]	AP Diameter (mm)
	IC_D_R	[0.97, 1.29]	Intercommissural Diameter Ratio (unitless)
	AL_R	[3.2, 7.2]	Anterior Leaflet Length Ratio (unitless)
	PL_R	[0.16, 1]	Posterior Leaflet Length Ratio (unitless)
	CL_R	[0.5, 0.75]	Commissure Length Ratio (unitless)
	Ant_Blunt	[0.5, 2.05]	Anterior Leaflet Bluntness (unitless)
	AH	[0.3, 0.7]	Anterior Height (mm)
	PH	[1, 2]	Posterior Height (mm)
	D_Shape	[0, 0.038]	D_Shape (unitless)
	SF_Shape_R	[0.01, 0.019]	Septal Flat Shape Ratio (unitless)
	Com_Loc	[0.26, 0.6]	Commissure Location (unitless)
	Com_Cur	[0.2, 0.6]	Commissure Curvature (unitless)
Papillary Muscles and Annulus BC	ALPM_dist	18.06	The relative location of papillary muscles was defined by 6 parameters (2 horizontal distance, 2 vertical distance, and 2 circumferential angles). (Units mm and deg)
	PMPM_dist	16.27	
	H_ALPM	24.975	
	H_PMPM	25.135	
	ALPM_Phi	118	
	PMPM_Phi	108	
	max_ant_length	0	Papillary muscle dynamics were defined by 3 parameters (vertical displacement of PMs and inter-PM displacement).
	max_pst_length	0	
	inter_PAP_du	5	
	PMPM_dz	3.5	
	ALPM_dz	3.5	
	Annulus_dh	1	Annulus motion during the cardiac cycle was defined by 2 parameters (change in saddle shape and radial expansion)
	Annulus_dr	2	

**Table 2:** Holzapfel–Ogden material parameters for anterior and posterior mitral valve leaflets. Parameters a, a_f_, a_s_, a_fs_ are expressed in MPa and b, b_f_, b_s_, b_fs_ are unitless.

	a	b	a_f_	b_f_	a_s_	b_s_	a_fs_	b_fs_
Anterior	7.5 × 10^−5^	14.447	0.092915	273.80	0.02239	75.32	0.0	1.0
Posterior	7.5 × 10^−5^	14.447	0.027282	130.48	0.00300	25.16	0.0	1.0

## Data Availability

The datasets used/analyzed are available from the corresponding author upon reasonable request.

## References

[1] Galusko V., Sekar B., Ricci F., Wong K., Bhattacharyya S., Mullen M., Gallina S., Ionescu A., Khanji M.Y. (2022). Mitral Regurgitation Management: A Systematic Review of Clinical Practice Guidelines and Recommendations. Eur. Heart J. Qual. Care Clin. Outcomes.

[2] Stone G.W., Lindenfeld J., Abraham W.T., Kar S., Lim D.S., Mishell J.M., Whisenant B., Grayburn P.A., Rinaldi M., Kapadia S.R. (2018). Transcatheter Mitral-Valve Repair in Patients with Heart Failure. N. Engl. J. Med..

[3] Feldman T., Wasserman H.S., Herrmann H.C., Gray W., Block P.C., Whitlow P., St. Goar F., Rodriguez L., Silvestry F., Schwartz A. (2005). Percutaneous Mitral Valve Repair Using the Edge-to-Edge Technique. J. Am. Coll. Cardiol..

[4] Mauri L., Foster E., Glower D.D., Apruzzese P., Massaro J.M., Herrmann H.C., Hermiller J., Gray W., Wang A., Pedersen W.R. (2013). 4-Year Results of a Randomized Controlled Trial of Percutaneous Repair Versus Surgery for Mitral Regurgitation. J. Am. Coll. Cardiol..

[5] Sugiura A., Kavsur R., Spieker M., Iliadis C., Goto T., Öztürk C., Weber M., Tabata N., Zimmer S., Sinning J.-M. (2022). Recurrent Mitral Regurgitation After MitraClip: Predictive Factors, Morphology, and Clinical Implication. Circ. Cardiovasc. Interv..

[6] Dal-Bianco J.P., Levine R.A. (2013). Anatomy of the Mitral Valve Apparatus. Cardiol. Clin..

[7] Kreidel F., Zaid S., Tamm A.R., Ruf T.F., Beiras-Fernandez A., Reinold J., Geyer M., da Rocha e Silva J., Schnitzler K., Michaela H. (2021). Impact of Mitral Annular Dilation on Edge-to-Edge Therapy With MitraClip-XTR. Circ. Cardiovasc. Interv..

[8] Krawczyk-Ożóg A., Hołda M.K., Sorysz D., Koziej M., Siudak Z., Dudek D., Klimek-Piotrowska W. (2017). Morphologic Variability of the Mitral Valve Leaflets. J. Thorac. Cardiovasc. Surg..

[9] Zamorano J.L., González-Gómez A., Lancellotti P. (2014). Mitral Valve Anatomy: Implications for Transcatheter Mitral Valve Interventions. EuroIntervention.

[10] Kim J., Palumbo M.C., Khalique O.K., Rong L.Q., Sultana R., Das M., Jantz J., Nagata Y., Devereux R.B., Wong S.C. (2019). Transcatheter MitraClip Repair Alters Mitral Annular Geometry—Device Induced Annular Remodeling on Three-Dimensional Echocardiography Predicts Therapeutic Response. Cardiovasc. Ultrasound.

[11] Schueler R., Momcilovic D., Weber M., Welz A., Werner N., Mueller C., Ghanem A., Nickenig G., Hammerstingl C. (2014). Acute Changes of Mitral Valve Geometry During Interventional Edge-to-Edge Repair With the MitraClip System Are Associated With Midterm Outcomes in Patients With Functional Valve Disease. Circ. Cardiovasc. Interv..

[12] Katz W.E., Conrad Smith A.J., Croc F.W., Cavalcante J.L. (2017). Echocardiographic Evaluation and Guidance for MitraClip Procedure. Cardiovasc. Diagn. Ther..

[13] Dabiri Y., Mahadevan V.S., Guccione J.M., Kassab G.S. (2022). A Simulation Study of the Effects of Number and Location of MitraClips on Mitral Regurgitation. JACC Adv..

[14] Mansi T., Voigt I., Georgescu B., Zheng X., Mengue E.A., Hackl M., Ionasec R.I., Noack T., Seeburger J., Comaniciu D. (2012). An Integrated Framework for Finite-Element Modeling of Mitral Valve Biomechanics from Medical Images: Application to MitralClip Intervention Planning. Med. Image Anal..

[15] Stevanella M., Votta E., Redaelli A. (2009). Mitral Valve Finite Element Modeling: Implications of Tissues’ Nonlinear Response and Annular Motion. J. Biomech. Eng..

[16] Liu H., Simonian N.T., Pouch A.M., Iaizzo P.A., Gorman J.H., Gorman R.C., Sacks M.S. (2023). A Computational Pipeline for Patient-Specific Prediction of the Postoperative Mitral Valve Functional State. J. Biomech. Eng..

[17] Sacks M.S., Drach A., Lee C.-H., Khalighi A.H., Rego B.V., Zhang W., Ayoub S., Yoganathan A.P., Gorman R.C., Gorman J.H. (2019). On the Simulation of Mitral Valve Function in Health, Disease, and Treatment. J. Biomech. Eng..

[18] Caballero A., Mao W., McKay R., Hahn R.T., Sun W. (2020). A Comprehensive Engineering Analysis of Left Heart Dynamics After MitraClip in a Functional Mitral Regurgitation Patient. Front. Physiol..

[19] Lazarus A., Dalton D., Husmeier D., Gao H. (2022). Sensitivity Analysis and Inverse Uncertainty Quantification for the Left Ventricular Passive Mechanics. Biomech. Model. Mechanobiol..

[20] Thiel J.-N., Gestrich J., Steinseifer U., Friehs I., Diaz-Gil D., Neidlin M. (2025). Quantifying the Impact of Mitral Valve Anatomy on Clinical Markers Using Surrogate Models and Sensitivity Analysis. Comput. Biol. Med..

[21] Tondi D., Sturla F., Marin-Cuartas M., Dieterlen M.-T., Jahnke C., Paetsch I., Spampinato R.A., Borger M.A., Votta E. (2026). Magnetic Resonance-Based Computational Modelling of Healthy and Prolapsing Mitral Valves to Quantify the Load Transfer between the Mitral Apparatus and the Ventricular Myocardium. Comput. Methods Programs Biomed..

[22] Munafò R., Saitta S., Vicentini L., Tondi D., Ruozzi V., Sturla F., Ingallina G., Guidotti A., Agricola E., Votta E. (2025). MitraClip Device Automated Localization in 3D Transesophageal Echocardiography via Deep Learning. Comput. Methods Programs Biomed..

[23] Dabiri Y., Yao J., Mahadevan V.S., Gruber D., Arnaout R., Gentzsch W., Guccione J.M., Kassab G.S. (2021). Mitral Valve Atlas for Artificial Intelligence Predictions of MitraClip Intervention Outcomes. Front. Cardiovasc. Med..

[24] Kamakoti R., Dabiri Y., Wang D.D., Guccione J., Kassab G.S. (2019). Numerical Simulations of MitraClip Placement: Clinical Implications. Sci. Rep..

[25] Pham T., Sun W. (2014). Material Properties of Aged Human Mitral Valve Leaflets. J. Biomed. Mater. Res. A.

[26] Holzapfel G.A., Ogden R.W. (2009). Constitutive Modelling of Passive Myocardium: A Structurally Based Framework for Material Characterization. Philos. Trans. R. Soc. A Math. Phys. Eng. Sci..

[27] Rasmussen C.E., Williams C.K.I. (2005). Gaussian Processes for Machine Learning.

[28] Sobol′ I.M. (2001). Global Sensitivity Indices for Nonlinear Mathematical Models and Their Monte Carlo Estimates. Math. Comput. Simul..

[29] Kostelnik C.J., Meador W.D., Lin C.-Y., Mathur M., Malinowski M., Jazwiec T., Malinowska Z., Piekarska M.L., Gaweda B., Timek T.A. (2025). Tricuspid Valve Leaflet Remodeling in Sheep with Biventricular Heart Failure: A Comparison between Leaflets 2024. Acta Biomater..

[30] Haese C.E., Dubey V., Mathur M., Pouch A.M., Timek T.A., Rausch M.K. (2025). Tricuspid Valve Edge-to-Edge Repair Simulations Are Highly Sensitive to Annular Boundary Conditions. J. Mech. Behav. Biomed. Mater..

[31] Praz F., Borger M.A., Lanz J., Marin-Cuartas M., Abreu A., Adamo M., Ajmone Marsan N., Barili F., Bonaros N., Cosyns B. (2025). 2025 ESC/EACTS Guidelines for the Management of Valvular Heart Disease. Eur. Heart J..

[32] Grayburn P.A., Thomas J.D. (2021). Basic Principles of the Echocardiographic Evaluation of Mitral Regurgitation. JACC Cardiovasc. Imaging.

